# The Role of Everolimus in Renal Cell Carcinoma

**DOI:** 10.15586/jkcvhl.2015.43

**Published:** 2015-12-30

**Authors:** Malek Meskawi, Roger Valdivieso, Paolo Dell’Oglio, Vincent Trudeau, Alessandro Larcher, Pierre I. Karakiewicz

**Affiliations:** 1 Cancer Prognostics and Health Outcomes Unit, University of Montreal Health Center, Montreal, Quebec, Canada; 2 Division of Oncology, Unit of Urology, URI, IRCCS Ospedale San Raffaele, Milan, Italy.

**Keywords:** everolimus, kidney cancer, mTOR, renal cell carcinoma

## Abstract

Everolimus (RAD001) is an orally administered agent that inhibits the mammalian target of rapamycin serine-threonine kinase. A phase III pivotal trial on everolimus, published in 2008, provided the first evidence for the efficacy of sequential therapy for patients with metastatic clear cell renal cell carcinoma (RCC). In this study, everolimus was used after failure of one or several previous lines of therapy, and it demonstrated a 3-month survival benefit relative to placebo. Currently, based on the level 1 evidence, everolimus represents the molecule of choice for third-line therapy after failure of previous two tyrosine kinase inhibitors (TKIs). However, second-line use after failure of one TKI is challenged by two new molecules (nivolumab and cabozantinib), which proved to have better efficacy with similar toxicity profile. In non-clear cell metastatic RCC, the current evidence recommends everolimus as a second-line therapy after failure of previous first-line sunitinib.

## Introduction

Renal cell carcinoma (RCC) is the seventh leading cancer among men and the tenth among women in the United States. In 2015, an estimated 61,560 new cases of RCC were diagnosed in the United States (1).The pathogenesis of the dominant clear cell histological subtype of RCC is associated with a loss of the von Hippel-Lindau (VHL) gene function on chromosome 3p. The VHL tumor suppressor gene codes for VHL protein that regulates cellular response to hypoxia by targeting hypoxia-inducible factor. Inactivation of VHL leads to an increased blood vessel formation through the upregulation of angiogenic factors, such as vascular endothelial growth factor (VEGF), vascular endothelial growth factor receptor (VEGFR), platelet-derived growth factor receptor, and epidermal growth factor receptor (2).

Surgical treatment represents the standard of care in the management of localized RCC. However, up to 16% of patients present with de novo distant metastases at diagnosis, and about 30% of patients eventually develop metastases during follow-up (3). Historically, the median survival of patients with metastatic RCC (mRCC) has been 10 months (4). The introduction of targeted therapies resulted in a paradigm shift in the management of this malignancy (5). The use of first-, second-, and subsequent-line targeted therapies resulted in up to 2-year increase in the life expectancy of patients with mRCC (6). Two subtypes of targeted therapies exist for the treatment of mRCC (tyrosine kinase inhibitor [TKI] and mammalian target of rapamycin inhibitors [mTORi]). Five TKIs (sunitinib, sorafenib, pazopanib, axitinib, and bevacizumab) and two mTORi (temsirolimus and everolimus) were approved for the treatment of mRCC (5, 7–12). Everolimus (RAD001) is an orally administered agent that inhibits the mTOR serine-threonine kinase. mTOR acts as a biological switch that regulates cellular metabolism, growth, and angiogenesis. In consequence, the disruption of mTOR pathway suppresses the progression of cancer cells through the inhibition of cell cycle and angiogenesis.

A phase III trial on everolimus, published in 2008, provided the first evidence for the efficacy of sequential therapy for patients with metastatic clear cell RCC, where everolimus was used after failure of one or several previous lines of therapy and demonstrated a 3-month survival benefit relative to placebo (13). The practice of sequential therapy has since then become the standard of care. Today, not only everolimus but several other molecules are available for use in sequential fashion. Based on the alternative for second-line therapy, the role of everolimus needs to be revised, and the objective of this review is to provide evidence supporting the optimal use of everolimus in the setting of metastatic clear cell RCC. To address this objective, evidence supporting its use either as first or subsequent line, as well as alone or in combination, is reviewed.

## Materials and methods

A comprehensive PubMed literature search was performed for articles published between 2007 and 2015 using the key words “everolimus,” “RAD001,” “kidney,” and “renal cell carcinoma” in the PubMed library up to September 2015. Moreover, abstracts presented at the American Society of Clinical Oncology (ASCO) and the European Society of Medical Oncology (ESMO) annual meetings between the years 2009 and 2015 were also retrieved. The search was limited to English literature, humans, and persons aged 18 years and older. The subject and outcome of interest, pertinence, quality, and details of reporting were the indicators of manuscript quality. Only data from phase II and III trials and expanded access program were included.

## Clinical efficacy of everolimus

### Data supporting sequential everolimus after failure of one or several previous treatment lines: RECORD-1 study

The efficacy of everolimus in the management of metastatic clear cell RCC refractory to one or several lines of previous systemic therapy was confirmed in a multi-institutional phase III, placebo-controlled trial (Renal Cell cancer treatment with Oral RAD001 given Daily [RECORD-1]). In this study, 410 patients were randomized to 10 mg/daily everolimus (n=272) or placebo and best supportive care (n=138). Most patients received multiple systemic agents that ranged from immunotherapy to cytotoxic chemotherapy prior to randomization with everolimus or placebo. The median progression-free survival (PFS) for everolimus was statistically superior to that for placebo and best supportive care (4.9 vs. 1.9 months; Hazard ration [HR]: 0.33; P<0.001) (13). These data provided the first prospective and placebo-controlled evidence for the use of everolimus as a sequential therapy. Recommendation for specific second-line sequencing of this molecule was made upon publication (14). Its rationale was based on the lack of alternative data for specific second-line therapy from within randomized designs.

Two additional features of this pivotal trial deserve mention (13, 15). First, less than 5% received first-line VEGF therapy that would reflect contemporary first-line use. In 46 patients who were randomized to everolimus or placebo after sunitinib failure, the recorded PFS benefit was 4.6 vs. 1.8 months. Second, as many as 26% of patients received two prior lines of VEGF-TKIs (sorafenib and sunitinib). Here, the benefit of everolimus given in second line was 5.4 vs. 1.9 months, relative to placebo. Unfortunately, the study included a marginal proportion of patients in whom everolimus was delivered in a second-line setting, and even fewer specifically received first-line sunitinib. Nonetheless, the study provided excellent third-line and subsequent-line efficacy for everolimus because the vast majority (79%) of enrolled patients received multiple previous lines (16).

The pivotal trial results were corroborated by population-based data from the everolimus expanded access program RAD001 expanded access clinical trial (REACT), where everolimus was offered to patients who progressed during initial VEGFR-TKI. In this study (n=1,367), 38.5% of patients received only one prior VEGFR-TKI, whereas 31.6% received two prior VEGFR-TKI lines. In the REACT study, 51.6 and 1.7% of patients treated with everolimus, respectively, achieved stable disease or partial remission at follow-up. Finally, 23.7% of patients progressed on everolimus (17).

### How to interpret the place of sequential everolimus in the era of the AXIS trial

The findings of the RECORD-1 study, published in August 2008, revolutionized the management of mRCC (13). Specifically, they introduced the notion of sequential therapy and validated the efficacy of everolimus in this setting. In December 2011, Rini et al. (11) reported a phase III study comparing two alternative second-line therapies, axitinib vs. sorafenib, after failure of a single first-line therapy (sunitinib, cytokine, bevacizumab, and temsirolimus). Because of its design, the AXIS study distinguished itself from the RECORD-1 study. First and foremost, the sequential nature of the AXIS study design deserves mention: the sequencing was specifically and exclusively defined as second line. In contrast, in the RECORD-1 study, only 21% of patients received everolimus as second line. Second, a smaller proportion received a targeted therapy as in first line and even fewer received first-line sunitinib, the established contemporary standard of care in first-line therapy (National Comprehensive Cancer Network [NCCN]). The vast majority of patients in the RECORD-1 study (79%) received everolimus as third or subsequent lines. In the AXIS study, all patients received sequential second-line therapy. Moreover, in the AXIS study, the majority of patients (54%) received first-line sunitinib, which is consistent with guideline recommendations and reflects the standard of care in clinical practice. Finally, it is of note that the potential benefit of an alternative mode of action (mTOR inhibition) of everolimus after failure of first-line VEGF-based therapy does not appear to represent an important argument favoring everolimus relative to a second-line VEGF-based therapy, such as axitinib. The relatively comparable median PFS of everolimus in second line vs. axitinib after sunitinib failure and numerous data from sequential VEGF-use studies, where the efficacy of sequential VEGF was confirmed, clearly support this contention (11, 18).

In summary, robust data based on 79% of the RECORD-1 study cohort support the use of everolimus in third or subsequent lines. Conversely, the AXIS-1 study strongly supports the use of axitinib as second line, especially after failure of sunitinib. The sequence of first-line sunitinib, followed by second-line axitinib and eventually followed by third-line everolimus, appears most justified according to the existing data. Nonetheless, existing guidelines do not exclude the possibility of relying on second-line everolimus.

### Efficacy of everolimus as in first-line therapy: RECORD-3 study

Based on the encouraging efficacy and tolerability of everolimus after failure of previous lines, a randomized, phase II noninferiority study was launched with the intent of challenging the established first-line role of sunitinib in the setting of metastatic clear cell RCC (6). Specifically, the design postulated noninferiority of median combined PFS and/or of overall survival (OS) with the use of first-line everolimus followed by second-line sunitinib (n=238 vs. the opposite sequence n=233). The majority of patients included displayed clear cell histological subtype (85%) and favorable or intermediate Memorial Sloan Kettering Cancer Center (MSKCC) prognosis (85%). The combined PFS medians were 21.1 vs. 25.8 months for the everolimus-sunitinib vs. sunitinib-everolimus arm (HR: 1.3; 95% confidence interval [CI]: 0.9–1.7), respectively. The combined OS medians were 22.4 vs. 32.0 months for the everolimus-sunitinib vs. sunitinib-everolimus arm (HR: 1.2; 95% CI: 0.9–1.6), respectively. In consequence, the noninferiority of the proposed PFS and/or OS sequence of everolimus followed by sunitinib could not be supported. Therefore, the RECORD-3 data do not support the use of everolimus as first line instead of the established standard of care, sunitinib.

### First- or second-line everolimus combination therapy with bevacizumab: RECORD-2 study

The concept of combining two agents with different mechanisms of action is attractive because it may offer greater efficacy and PFS. This premise was used to test the efficacy and tolerability of combined everolimus and bevacizumab (VEGF inhibitor) as either first-line or second-line therapies. Hainsworth et al. (19) addressed this hypothesis in a phase II study. Here, 50 previously untreated and 30 treated patients were administered a combination of first-line everolimus and bevacizumab. The median first-line PFS was 9.1 months, the median second-line PFS was 7.1 months, and toxicity profiles were very favorable. Based on these promising data, two large studies were designed to test this regimen (20). One was a large first-line, randomized phase II study comparing everolimus and bevacizumab regimen to bevacizumab and interferon regimen (RECORD-2 study). The other was a second-line, phase III post-sunitinib study comparing the same regimen to everolimus plus placebo.

The phase II, open-label RECORD-2 trial randomized 365 treatment-naive mRCC patients to bevacizumab 10 mg/kg every 2 weeks plus everolimus 10 mg/day or bevacizumab plus interferon alpha-2a 3–9 MU three times/week. All patients had prior nephrectomy, and more than 90% had a good-to-intermediate MSKCC performance status. There was no significant difference between everolimus and interferon groups in objective response rates (27 vs. 28%) or median PFS based on central review (9.3 months vs. 10; HR: 0.91; P=0.485) (20). The second study is still ongoing. It aims to compare the efficacy of everolimus combined with bevacizumab versus everolimus alone in second line after failure of previous sunitinib in patients with mRCC (NCT01198158).

### Head-to-head comparison in second- and subsequent-line use of everolimus

In a phase II trial, Jonasch et al. (21) compared MK-2206, a selective inhibitor of phosphoinositide-3-phosphate kinase (PI3K), with everolimus in patients who had failed one or two prior VEGF inhibitors (n=43). Their results showed an inferior median PFS for MK-2206 compared to everolimus (3.65 vs. 7.43 months). Similarly, Powles et al. (22) compared the efficacy of second-line use of a PI3K/mTOR inhibitor (GDC-0980) to everolimus in patients with clear cell mRCC (n=85). Their analysis revealed that GDC-0980 was inferior to everolimus (median PFS: 3.7 vs. 6.1 months; HR: 2.04; P<0.01).

Two comparative phase III trials, aimed at exploring everolimus use in patients with clear cell mRCC who received one or two prior antiangiogenic agents, have been published recently (23, 24). The first study (METEOR) compared the efficacy of cabozantinib to everolimus in second and subsequent line after failure of previous TKI or cytokine therapy. Cabozantinib is a TKI that targets VEGFR, MET, and AXL. The latter two are associated with increased resistance to VEGFR inhibitors. The PFS and the interim OS analyses favored cabozantinib relative to everolimus. Specifically, the median PFS was 7.4 months for cabozantinib vs. 3.8 months for everolimus (HR: 0.58; 95% confidence interval [CI]: 0.45–0.75; P<0.001). Both the molecules presented similar toxicity profiles. It is of note that 73% of patients were treated in second line and the remaining 27% represented third-line patients. In subgroup analyses, patients treated with second-line cabozantinib showed better median PFS relative to everolimus (HR: 0.56; 95% CI: 0.42–0.75). A difference in PFS, albeit a nonsignificant one, was shown in individuals who represented third-line patients (HR: 0.67; 95% CI: 0.41–1.1) (23). This observation implies that cabozantinib is better than everolimus in second but not necessarily in third line.

The second phase III study compared nivolumab with everolimus in patients with clear cell mRCC, who were previously treated with one or two TKIs (sunitinib, pazopanib, and axitinib). Nivolumab is a human programmed death 1 (PD-1) receptor antibody that disrupted PD-1 and PD-ligand 1 (PD-L1) signaling pathway, which resulted in increased antitumor immunity (24). The majority of study cohort (78%) represented second-line patients. The remainder represented third-line patients. The median OS was 25 months for nivolumab compared to 19.6 months for everolimus (HR: 0.73; P=0.002). However, no statistically significant differences were observed for median PFS between both arms (4.6 for nivolumab vs. 4.4 months for everolimus; HR: 0.88; P=0.11). This observation implies that nivolumab may improve responses to subsequent agents, without demonstrating immediate PFS advantage relative to everolimus. Taken together, both studies show that novel molecules might provide better PFS or OS outcomes relative to everolimus.

### Data supporting the use of sequential everolimus after mTOR failure

No level 1 evidence supports the use of sequential everolimus after failure of previous mTORi (temsirolimus). Indeed, previous temsirolimus use was an exclusion criterion for phase III trials. Maj-Hes et al. (25) reported, in a retrospective study, on the use of everolimus as third or fourth line after failure of previous one or two TKI and one mTORi (temsirolimus). Seven patients were included in this study. The median PFS duration for everolimus after failure of previous temsirolimus was 5.8 months.

### Efficacy of everolimus in non-clear cell mRCC

Temsirolimus represents the standard of care for non-clear cell mRCC. Based on its established efficacy (9), Koh et al. (26) explored the efficacy of everolimus in patients with non-clear cell RCC. In their single-arm phase II trial (n=49), 59.2% (n=29) of patients had papillary subtype and the remaining 16.3% (n=8) patients had chromophobe RCC. First-line everolimus was used in 53.1% (n=26) of patients. In the remaining groups, everolimus was used as second line after the failure of prior sunitinib or sorafenib. In the first-line patients, everolimus use resulted in a median PFS of 3.7 months, whereas it was 5.3 months in the second line.

Because the prognosis of patients with papillary mRCC was better than that of patients with other histological subtypes, a phase II study (RAPTOR) was designed to investigate the efficacy of first-line everolimus in patients with papillary mRCC. In this study (n=92), the median OS for first-line everolimus use was 21.1 months. Specifically, the median OS for types 1 and 2 papillary mRCC were 28 and 20.3 months, respectively (27).

Recently, a randomized phase II trial (ESPN) compared the efficacy of first-line everolimus followed by second-line sunitinib versus first-line sunitinib followed by second-line everolimus in patients with non-clear cell mRCC (n=68). The majority were good or intermediate MSKCC risk. The median PFS for first-line sunitinib was 6.1 vs. 4.1 months for first-line everolimus (P=0.25). Of all, 39 patients received second-line therapy. The median PFS for second-line sunitinib was 1.8 vs. 4.3 months for second-line everolimus. The median OS recorded in patients exposed to first-line everolimus (10.5 months) was inferior to the OS recorded in patients exposed to first-line sunitinib (median OS not reached; P=0.01). In consequence, based on the OS inferiority recorded in the arm exposed to first-line everolimus and second-line sunitinib, the study was terminated (28).

Finally, an ongoing phase II trial compares the efficacy of first-line everolimus vs. sunitinib in patients with non-clear cell mRCC (ASPEN trial). In this study, patients were stratified according to histological subtypes (papillary, chromophobe, and unclassified) and MSKCC risk score (good, intermediate, and poor). The preliminary results showed that everolimus was better than sunitinib in patients with chromophobe histology (11.4 vs. 5.5 months) or poor MSKCC risk (6.1 vs. 4 months). Conversely, sunitinib was the preferred first-line molecule for papillary, unclassified histology, as well as for patients with good or intermediate MSKCC risk score (29).

Based on the current evidence, everolimus is recommended as the second-line treatment in patients with non-clear cell mRCC after failure of previous first-line sunitinib. More studies are warranted to test its efficacy after failure of first-line temsirolimus in the non-clear cell mRCC setting.

## Safety and tolerability

Because patients with metastatic disease have limited life expectancy, assessment of everolimus toxicities is essential before treatment administration. The most common clinical toxicities in patients treated with everolimus for mRCC were stomatitis (44%), infections (37%), asthenia (33%), rash (29%), fatigue (31%), diarrhea (30%), and anorexia (25%). Of these, infectious complications (10%), stomatitis (4%), fatigue (5%), and pneumonitis (4%) represented G3-4 toxicities. The most frequent laboratory toxicities consisted of hypercholesterolemia (77%), anemia (92%), and hyperglycemia (57%). Of these, lymphopenia (18%), hyperglycemia (12%), and anemia (13%) represented G3-4 toxicities (10). Comparable toxicity rates were found across all studies that assessed the clinical efficacy of everolimus in first or subsequent line in patients with mRCC (23, 24). It should be noted that everolimus-associated pneumonitis, stomatitis, as well as increased cholesterol and triglycerides levels, predicted better survival outcomes in patients with mRCC compared to patients who did not experience these adverse events: pneumonitis (median OS: 15.4 vs. 7.4 months; P<0.001; HR: 0.32), stomatitis (median OS: 30.6 vs. 14 months; P=0.004), and hyperlipidemia (median OS: 26.4 vs. 13.4 months; P=0.018) (30).

Another important consideration in patients with mRCC is the change in patients’ quality of life after everolimus use. Using the everolimus phase III data, Beaumont et al. (31) used the Functional Assessment of Cancer Therapy Kidney Symptom Index- disease related symptoms (FKSI-DRS) and the European Organization for Research and Treatment of cancer (EORTC) QLQC30 as tools to assess patients’ quality of life before and after everolimus administration. They showed that patients treated with everolimus had comparable quality of life and physical functioning relative to patients treated with placebo and best supportive care.

## Conclusion

Everolimus is the standard second- or third-line therapy in patients with clear cell or non-clear cell mRCC who failed prior VEGFR-TKI. However, its use in first line is not supported by evidence. Everolimus is well tolerated and is known for favorable acceptable rate of adverse events. Its place in the treatment paradigm of mRCC might be challenged by two emerging molecules (cabozantinib and nivolumab), which are tested in phase III trials. Moreover, comparative studies with the other second-line standard of care, for example, axitinib, are needed.
